# Automated annotation of functional imaging experiments via multi-label classification

**DOI:** 10.3389/fnins.2013.00240

**Published:** 2013-12-16

**Authors:** Matthew D. Turner, Chayan Chakrabarti, Thomas B. Jones, Jiawei F. Xu, Peter T. Fox, George F. Luger, Angela R. Laird, Jessica A. Turner

**Affiliations:** ^1^Department of Computer Science, University of New MexicoAlbuquerque, NM, USA; ^2^Mind Research NetworkAlbuquerque, NM, USA; ^3^Conjectural SystemsAtlanta, GA, USA; ^4^Research Imaging Center, University of Texas Health Science CenterSan Antonio, TX, USA; ^5^Department of Physics, Florida International UniversityMiami, FL, USA; ^6^Department of Psychology and Neuroscience Institute, Georgia State UniversityAtlanta, GA, USA

**Keywords:** text mining, data mining, multi-label classification, bioinformatics, CogPO, neuroimaging, annotations

## Abstract

Identifying the experimental methods in human neuroimaging papers is important for grouping meaningfully similar experiments for meta-analyses. Currently, this can only be done by human readers. We present the performance of common machine learning (text mining) methods applied to the problem of automatically classifying or labeling this literature. Labeling terms are from the Cognitive Paradigm Ontology (CogPO), the text corpora are abstracts of published functional neuroimaging papers, and the methods use the performance of a human expert as training data. We aim to replicate the expert's annotation of multiple labels per abstract identifying the experimental stimuli, cognitive paradigms, response types, and other relevant dimensions of the experiments. We use several standard machine learning methods: naive Bayes (NB), *k*-nearest neighbor, and support vector machines (specifically SMO or sequential minimal optimization). Exact match performance ranged from only 15% in the worst cases to 78% in the best cases. NB methods combined with binary relevance transformations performed strongly and were robust to overfitting. This collection of results demonstrates what can be achieved with off-the-shelf software components and little to no pre-processing of raw text.

## 1. Introduction

Scientific publication in cognitive neuroscience today is proceeding at an intense pace; a pubmed.gov search revealed that for the 4 year period 2009–2012, there were 5033 total publications tagged “human brain mapping,” with the number of publications between 2009 and 2012 increasing by 12% each year. The situation is similar in other fields. We are faced with a deluge of new results and publications across all fields every year (Howe et al., 2008). This has created problems for data warehousing, searching, and curation. This latter term refers to the acquisition, selection, annotation, and maintenance of digital information.

The curation of this massive collection of scientific literature is a challenging problem. Although some tools exist to assist researchers with the management of this vast collection of data, most curation of scientific research literature is done in-house by the researchers themselves. Among the primary tools of curation are computer ontologies and controlled vocabularies (Trieschnigg et al., 2009). Controlled vocabularies limit language to terms with precise unitary meanings and ontologies replicate some of the logical structure of scientific language in a computable fashion, allowing researchers to more effectively search and process the scientific literature.

The BrainMap database (www.brainmap.org) was developed to provide a repository of the results from the human neuroimaging literature (Fox and Lancaster, 2002; Fox et al., 2005; Laird et al., 2005b; Lancaster et al., 2005; Turner and Laird, 2012). The BrainMap schema developed as a way to describe PET and fMRI experiments and the conditions which led to the activation loci reported in the publications. This schema describes the subject groups included in the analyses (e.g., healthy controls and adults with autism), the context of the experiment (e.g., a pre/post treatment study), the behavioral domain being studied by each analysis (e.g., attention and memory), the specific paradigm class (e.g., memory for faces), and a set of terms and relationships for the experimental stimuli used in the conditions being contrasted in each analysis. The terms used to describe the experimental conditions, their definitions and relationships, have been formalized in the Cognitive Paradigm Ontology (CogPO; Turner and Laird, 2012). The CogPO ontology can be downloaded as an OWL file from www.cogpo.org where additional information can be found.

The primary descriptors in CogPO are a set of terms for the Stimulus Type (e.g., flashing checkerboard, tone, word, or picture), the Stimulus Modality (e.g., visual, auditory, interoceptive), the Instructions given to the subject (e.g., attend, discriminate, imagine), and the Response Type (e.g., button press, speech) and Response Modality (the part of the body used to make the response, e.g., hand, foot, face). Each experimental condition is a combination of these characteristics, and the loci of activation are commonly the result of comparing fMRI BOLD signal during one combination versus another (for instance, changing the stimulus type or changing the instructions while maintaining the same stimuli). The BrainMap project includes the database of papers and experiments as well as related software to both to find papers based on these terms (Sleuth) and to perform meta-analysis over the results from comparable experiments (GingerALE). This toolset has led the way in meta-analyses of fMRI and PET studies, identifying commonalities of brain activation across the literature on working memory, depression, and many other topics (Farrell et al., 2005; Laird et al., 2005a; Fitzgerald et al., 2006; Menzies et al., 2008; Laird et al., 2009; Bzdok et al., 2012). The current database includes manually annotated results from approximately 2298 publications—covering 10,924 experiments—and spanning the last 20 years of human neuroimaging research.

While these manual curation methods are useful, there is a bottleneck; given the rate of publication it is challenging the for curators to manually annotate the literature as it is produced. Coupled with this is the fact that there are very few people in the scientific community whose primary task is curation, and they are often lacking in the specialized knowledge required for making classifications using the specialized terms. Lastly, the scientists producing the literature themselves are often neither qualified to annotate their own work nor are they interested in the annotation task *per se* (Lok, 2010). A technological solution appears to be required and will require the use of machine learning tools.

The problem of ontology annotation, the marking up of scientific articles with terms and semantic structure based on an ontology, is related to a machine learning problem known as “multi-label classification.” This is the most general form of the document classification task. The simplest form, binary classification, is the most well-developed area of automatic classification. In this task, learning machines are trained to determine if an instance (article) should be classified as being in a given class or not. We may think of this as determining if the instance has a label or does not; for instance, an article's content might be classified as “human brain mapping” or it might not. We are concerned with one choice and two options, either in the class or not in the class. Multi-*class* classification involves a set of classes that are mutually exclusive (every instance is in *at most* one class) and exhaustive (every instance is in *at least* one class). Here we are again concerned with a single choice, but there are more than two options. For instance, a newspaper article might be selected to be placed in the “sports,” “business,” or “local” section of the newspaper; each article to be printed must go into at least one section, and will appear in at most one section.

In multi-label classification, each instance classified will have some labels applied to it; the set of labels is not necessarily mutually exclusive or collectively exhaustive, and *a priori* we do not know which or how many labels a given instance may receive. An example of this is a newspaper's website. While articles can appear only in one section of a printed paper, on the website an article may be tagged with several sections. So an article on the financial situation of a sports team may be labeled “sports” and “business” and a story about a local restaurant sponsoring a local high-school football team might very well be labeled “business,” “local,” “sports,” and “food.” Binary and multi-class classification can be considered as special cases of, or restrictions on, the multi-label problem. The multi-label problem has been growing in importance as the internet has made larger pools of content available with no single classification scheme. For an overview of multi-label classification, see Tsoumakas and Katakis (2007) and Tsoumakas et al. (2010); for an overview of the technical issues involved, see Madjarov et al. (2012).

Recently there has been an increase in the application of machine learning methods to biomedical literature analysis. Many of these approaches seek novel algorithms to solve these problems. However, the machine learning literature is replete with well-established relatively simple methods for binary and multi-class problems that perform quite well. Additionally, there are a number of methods to transform multi-label problems into one of these more restrictive forms described above. Before developing entirely new algorithms, it is reasonable to ask whether or not the tools at hand can achieve useful results or if the increases in complexity that come with most new algorithms is worth the additional cost (Hand, 2006). Additionally, the application of these simpler methods may indicate where the issues in multi-label biomedical classification lie.

We seek to establish a baseline point of comparison for methods that may be developed for automated annotation of research abstracts using neuroimaging experimental terms. Here we apply entirely off-the-shelf solutions to the task of classifying scientific abstracts using the CogPO ontology. We present the methods in more detail than is perhaps common in the text-mining community, in service of making these results more repeatable by others, and to present these methods to neuroimaging researchers interested in automated annotation who may not otherwise be aware of them. The performance characteristics here may be viewed as a reasonable minimum performance point, which must be exceeded by new or more complex algorithms if they are to be viable competitors for practical applications in this arena.

## 2. Materials and methods

### 2.1. Data

The primary corpus consists of components of the bibliographic records, for 247 biomedical studies, retrieved from PubMed. These are based on a selection of studies examining differential brain activation patterns across an array of tasks in four high-profile mental disorders: schizophrenia, bipolar disorder, major depressive disorder, and autism spectrum disorders. These disorders were selected both for their importance and because they include larger samples of cognitive neuroimaging data. Each abstract was from a paper annotated using seven label dimensions: Behavioral Domain, Cognitive Paradigm, Instruction Type, Response Modality, Response Type, Stimulus Modality, and Stimulus Type. The label dimensions were not otherwise constrained; these are discussed in section 2.1.2.

#### 2.1.1. Corpora

The 247 PubMed records are basis for the training and testing instances for the machine learning algorithms. The features or attributes to be used for classification were vectors indicating the presence or absence of certain words in the abstract text, paper titles, Medical Subject Heading (MeSH) terms, or various combinations of these. (Note that the MeSH labels were limited to the “descriptor names” without the “qualifier names.”) There were five corpora used:

1. **Abstract Alone**. The text of each paper's abstract.
2. **Title Alone**. The words in the title of each paper.
3. **Keyword Alone**. The MeSH keywords for each paper.
4. **Title and Keyword**. The title words and MeSH keywords for each paper.
5. **Abstract, Title, and Keyword**. The text of each paper's abstract with MeSH keywords and article title words.

A final corpus that we mention in passing for completeness consisted of the abstract, title, and keyword corpus, passed through the NCBO annotator (bioportal.bioontology.org/annotator) to add annotations from several ontologies (not including CogPO) to determine if these markups would improve CogPO classification performance. The ontologies used for annotation were the Foundation Model of Anatomy (Rosse and Mejino, 2003), Cognitive Atlas (Poldrack et al., 2011), NIFSTD (Bug et al., 2008), and RadLex (Langlotz, 2006). The goal was to annotate the brain areas, other cognitive terms, or imaging methods that might have been mentioned in the abstract text. The NCBO Annotator leverages the structure of the NCBO ontologies to annotate text with generalizations of matching terms; if a word in the text being annotated matches a term in an ontology, the Annotator can also return the superclass(es) of the matching terms, to provide more general concepts. The ontologies used here were often very flat, though, without many levels available in the hierarchy (i.e., the immediate superclass was the root term), and thus only terms from the level matching the abstract text was included. There is a substantial overlap between the ontology annotator's results and the previously applied MeSH headings and base vocabulary of the abstracts; the dictionary for the annotated corpus included only one additional term. This Annotated corpus was also tested using the classifier algorithms but the performance was identical to the unannotated corpus, so we do not present the results.

The text was directly tokenized based on whitespace and punctuation, making each individual word into a token. This process also made numbers into tokens; the numbers were sometimes broken into multiple tokens (e.g., 0.5 became 0 and 5). No attempt was made to apply semantic mapping or concept identification to the original abstract text; each abstract word was treated as a single feature even when it should have been part of a multi-word token. Many of the MeSH labels and ontology annotations were also multi-word constructs, such as “Tomography, Emission-Computed.” In this case, we preserved the underlying concept by mapping these to single tokens. We were able to do this because the MeSH and ontology queries returned the multi-word concepts with explicit delimiters, allowing their preservation.

The text was then reduced by stopword removal, using the Natural Language Toolkit (NLTK; nltk.org) English stop word list (Loper and Bird, 2002; Bird et al., 2009). These were then converted to a “bag of words” vector representation with WEKA (Hall et al., 2009). Only the presence or absence, 1 or 0, respectively, of each word was recorded. In some applications, the term “bag of words” is reserved for vectors of counts; in this work the vectors are binary presence/absence representations. It should be noted that only basic English stop words were removed. No effort was made to remove numbers (meaningless in a “bag of words context”), specialized biomedical terminology occurring either too often or not often enough to be discriminating, and any other low-information vocabulary.

This produced for each corpus a collection of 247 instance vectors, one for each abstract, each of a length equal to the length of the dictionary for that corpus. Each of the corpora had a different dictionary length. The abstract alone corpus had a dictionary length of 3603 words; title alone had 670 words; keyword alone was 377 words; title and keyword was 999 words; and abstract, title, and keyword was 3918 words.

#### 2.1.2. Labels

The labels for each abstract came from the expert assignment of CogPO terms to the corresponding scientific papers as they are entered into the BrainMap database. CogPO provides a number of dimensions of labels, as described above in the BrainMap schema. We used the following dimensions: *behavioral domain*, *cognitive paradigm class*, *instruction type*, *response modality*, *response type*, *stimulus modality*, and *stimulus type*. The number of labels present in each dimension range from 5 to 48; see Table 1. The number of labels per dimension reported here are the numbers actually present in this particular sample of abstracts; CogPO has additional labels not used here in our available instances. Given our methods, labels without any instances would automatically drop out, so we can restrict the analysis to just the labels present without any loss of generality. For a complete listing of labels for these dimensions see: wiki.cogpo.org.

**Table 1:** **Characteristics of the data by dimension of the CogPO ontology and label sets**.

**Dimension**	# **Labels**	**LC_avg_**	**LC_max_**	**P_UNIQ_**	**P_min_**	***k***
Behavioral domain	40	1.846	8	0.429	0.413	9
Cognitive paradigm class	48	1.291	4	0.336	0.761	8
Instruction type	14	1.648	6	0.251	0.510	17
Response modality	5	1.308	3	0.036	0.700	21
Response type	9	1.324	4	0.069	0.696	10
Stimulus modality	5	1.150	3	0.036	0.858	25
Stimulus type	17	1.494	4	0.247	0.587	8

LC_avg_, LC_max_ = average and maximum number of labels per instance, respectively; P_UNIQ_ = ratio of unique label combinations/sample size (247); P_min_ = proportion with the minimum number of labels (always 1, in this dataset); k = value set for the kNN algorithm, see section 2.3.

Additional label characteristics presented in Table 1 are as follows. A standard measure in multi-label classification is label cardinality, the number of labels per instance. For multi-label data sets this varies by instance, and is usually reported as an average summary measure; here we present this usual average label cardinality as LC_avg_. We also include the maximum number of labels applied to a single instance, LC_max_; e.g., in the case of Behavioral Domain, at least one abstract was annotated with eight different terms, but the average number of labels was 1.846. The minimum (LC_min_) is always 1. The measure P_UNIQ_ for multi-label corpora is defined in Read et al. (2011), and is the number of unique label sets divided by the number of instances. Finally, P_min_, is the proportion of the data that is assigned the minimum number of labels, which for all of our dimensions is one label,

$${\text{P}}_{\text{min}}=\frac{\left|\left\{\text{Instances with 1 label}\right\}\right|}{N}$$

i.e., the number of instances with one label divided by the total number of instances. We use this measure instead of the P_max_ measure also defined in Read et al. (2011); in our case we felt this was more revealing. For our data P_max_ is always based on two cases (P_max_ = 0.0081; for both stimulus modality and response modality dimensions) or one case (0.0041; all other dimensions). Note that P_min_ shows that the modal number of labels for each dimension is 1; the median number of labels is 1 as well, for all dimensions, except for behavioral domain where it is 2.

### 2.2. Problem transformations

A problem transformation is any method that transforms multi-label data into a collection of single-label (binary) classification problems or which reduces a multi-label problem to a multi-class problem (Read et al., 2009; Tsoumakas et al., 2010; Cherman et al., 2011; Read et al., 2011; Santos et al., 2011; Modi and Panchal, 2012). Here we consider two problem transformation methods: binary relevance (BR) and label powerset (LP; also referred to as LC for “label concatenation”), which are the most common and well-researched. These methods are often implicitly incorporated into other methods. The benefit of abstracting out the transformations is that it allows new applications to be constructed easily by recycling binary and multi-class methods. In any use of a problem transformation method, both the transformation and the underlying classifier it is combined with must be indicated to have a complete specification.

Other problem transformation methods, not within the scope of this baseline analysis but certainly worth future consideration, include classifer chains (Read et al., 2009, 2011), pruned sets (Read et al., 2008), RAkEL (Tsoumakas et al., 2011); see (Santos et al., 2011) for a list. See Madjarov et al. (2012) for a substantial and recent review of this literature and comparison of the performance of many of these methods on other standard corpora.

*Notation:* Assume for the following that *L* is a set of labels for a given problem, |*L*| represents the size of the set *L* (i.e., number of labels), and λ stands in for an individual label as required. So, *L* = {λ_1_, λ_2_, …, λ_|*L*|_}. We let λ stand for the complement (negation) of λ. Following the literature, the set of instances will be called *D* and we will let *N* represent the number of instances in the training set, so: *N* = |*D*|. We let *d* represent the number of features of the feature space. Here *d* will equal the number of words in the dictionary and will vary by corpora.

#### 2.2.1. Binary relevance

The binary relevance (BR) method reduces a multi-label problem to collection of binary classification problems. It does this in the simplest and most obvious way; BR gives each label has its own classifier. For a problem with |*L*| labels, a separate classifier is built for each λ and, for a given classifier, each abstract is identified as either having the label λ or not, labeled λ. This reduces the |*L*|-label problem to |*L*| statistically independent binary problems, each with appropriately re-coded data. Therefore, any binary classifier may be applied to multi-label data.

For binary classifiers that produce probability or confidence estimates for each label, a threshold, *t* can be chosen for inclusion of that label in the multi-label classification of that instance. A threshold calibration procedure can be used to automatically select this value; a numerical grid search is conducted for values of *t* which match the average label cardinality of the predicted values for the test set to the average label cardinality to that of the training set for that fold (Fan and Lin, 2007; Read et al., 2011). The choice of *t* is not dependent on the accuracy of the predicted labels, just cardinality. If the average label cardinality for the training set is 2, for example, *t* is set so that the average label cardinality predicted for the testing set is as close to 2 as possible. This procedure is simple and efficient and empirically better justified than the arbitrary selection of a value for *t*. This procedure is applied in the cases of BR naive Bayes (NB) and BR *k*-nearest neighbor (kNN). NB returns probabilities for each label and *t* is set to a threshold probability, while kNN returns confidence values in the interval [0,1] and *t* is likewise used as a threshold. When BR is combined with sequential minimal optimization, the underlying algorithm returns only 1 or 0 for each label, so no thresholding is possible.

The problem with the BR method is clear: dependencies among the labels are ignored, as each is classified separately. However, the method is simple, both computationally and conceptually, and scales linearly with the number of labels |*L*| (Read et al., 2011); overall computational complexity will depend on the underlying classification algorithm. It is resistant to over-fitting, it does not require examples of every possible label combination and the models built for each label are independent of one another which allows updating of labels without having to completely recompute all the other models (Read et al., 2011). This is important for on-line or continually updating systems. Additionally, the assumption of independence among labels is similar to that made by NB regarding features (see below), and that method often works well-despite the assumption (Zhang, 2004, 2005). One would expect that more customized methods that can improve performance will make use of these dependencies.

#### 2.2.2. Label powerset

The label powerset (LP) method reduces a multi-label problem to a single multi-class problem. Under LP, each abstract's unique label combination is reduced to a single, corporate, label. With this method there will be as many labels as there are unique combinations. So an instance that is classified as λ_1_ and λ_2_ would receive the single combined label λ_12_. (We can assume a default label ordering on *L* such that λ_12_ and λ_21_ will be the same). Thus the collection of labels for each instance is reduced to a single label that is the concatenation of all the labels assigned to the instance.

For instance, for the behavioral domain label dimension we have 40 labels, appearing in 106 unique combinations (in Table 1, this number is P_UNIQ_ × 247 or P_UNIQ_ × *N*). From the point of view of the underlying classifier, this is a single classification with 106 mutually exclusive and exhaustive classes; each abstract is assigned to exactly one of the classes. Thus, any classifier that can be applied to a multi-class classification can be used. It is worth emphasizing that most binary classifiers have extensions to the multi-class problem already, so this transformation still allows a full range of off-the-shelf components to be used.

Under LP, a single classifier is built, and if this classifier assigns probabilities or confidences for each abstract to be assigned to each of the 106 unique combinations, then the single largest of these confidences is selected as the label combination. The underlying classifier simply reports the class selection, then that is used; there is no thresholding process as for BR.

Potential problems with this method are complexity and over-fitting. The computational complexity of this problem is a function of how the underlying learning algorithm handles the number of classes in a multi-class problem, but the worst case scales exponentially with |*L*|, although this is constrained by the amount of data, min(N, 2^|*L*|^−1), see Read et al. (2011) for details. However, for realistic cases this may be within a usable tolerance; our behavioral domain label set with 40 labels has a worst-case complexity of 10^12^, but both the number of actual label combinations (106) and size of the data set (247) severely restrict the problem to realistic computational requirements, here 10^2^ in either case. However, this matter is an empirical question and there may be data to which LP cannot reasonably be applied.

This method is very sensitive to the specific label combinations in the training data; it only learns the label combinations that are present, a kind of over-fitting. Thus, if new data are analyzed, with new label combinations not present in the training data, either the entire model will have to be retrained with new data, or the model without those combinations in the training data will never be able to specifically predict the new combinations.

### 2.3. Machine learning algorithms

Once a problem transformation has been applied to the data, a machine learning algorithm must be used on the transformed data. Here we consider three standard methods: Naive Bayes, *k*-nearest neighbor, and a type of support vector machine called sequential minimal optimization. These methods are relatively simple, easily available off the shelf, and are known to work well in a variety of machine learning and text mining contexts. The last two of these have hyperparameters that need to be chosen in order to evaluate their performance.

The *k* and *C* parameters were set once, at the start of the study, through exhaustive search using the entire data set, and with the log-loss criterion (Read et al., 2011) as a measure of performance and using the binary relevance transformation. This process was conducted before the data were broken into folds for performance testing and cross-validation, thus avoiding circularity or overfitting. For purposes of comparison, we run the algorithms with more or less optimized parameters, with the expectation of performance loss on real world data. For notation, see the beginning of section 2.2.

#### 2.3.1. Naive bayes

NB is a standard machine learning algorithm that is often used as a first approach for new problems (McCallum and Nigam, 1998; Eyheramendy et al., 2003; Rennie et al., 2003; Zhang, 2004; Witten et al., 2011); NB is often quite effective. The method uses Bayes' theorem to transform the label-conditional probabilities, *P*(feature | λ), derived from the training set, into *P*(λ | feature), the conditional probabilities of an instance having label λ given the presence of a feature. These probabilities, for each feature present in an instance, are combined to produce an estimate of the probability of the instance having label λ. The “naive” in the name refers to the assumption of feature independence present in the model. To make the calculations tractable, features are treated as statistically independent, usually an unreasonable assumption for real data. Mathematically this means that the probability of an instance having a label, *P*(λ) is the product of the *P*(λ | feature) values for features present in the abstract, and the compliments of these for features not present in a given abstract. Despite the logically unreasonable independence assumption, this method works quite well in most applications (McCallum and Nigam, 1998; Zhang, 2004, 2005), but see Rennie et al. (2003).

For binary classification, as under the BR method, the NB classifier for each label will return a probability for that label only. A threshold probability, *t*, can be chosen iteratively as described in section 2.2.1 on the BR transformation. For the LP method, a single NB classifier is built that returns a probability distribution across the unique label combinations. In this case, the label combination with the highest probability is chosen as the label combination for a new instance.

The NB classifier has no “tunable” hyperparameters affecting its performance. In that regard it is usually viewed as being data-driven.

#### 2.3.2. k-nearest neighbor

We implemented the *k*-nearest neighbor (kNN) classifier under both BR and LP; see Spyromitros et al. (2008) for a discussion of these methods. In kNN, the *k* nearest neighbors to the instance to be classified are found in feature space. For this to be meaningful, a definition of distance over the feature space must be adopted. We chose to use the Euclidian distance, as that tends to be a common default and is available off-the-shelf. Note that the distance between instances is computed in a very high dimensional space; each corpus' dictionary defines the dimension of the feature space. For example, in the abstract alone corpus, with 3603 tokens, the distances are computed between points in a 3603-dimensional feature space. This distance will be equal to the square root of the number of mismatched words in the two abstracts being compared; more mismatches means greater distance. Words not present in either abstract or words present in both do not affect the distance.

Once the *k* neighbors are found, their label frequencies are analyzed. Under the BR transformation, a confidence for each label is generated and a cutoff threshold, *t* is chosen as above. For the LP transformation, the most common unique label combination of the *k* neighbors is selected. For an alternate method that uses kNN internally, see Zhang and Zhou (2007).

The performance of kNN can be degraded by a variety of issues: noisy features, the presence of irrelevant features, or scaling of the feature values. The last of these is not a problem in our presence-absence approach (see section 2.1.1) as each feature is represented on the same scale. However, the large number of features surely presents many irrelevant features for classification, and there are terms used in vague, overlapping, and ambiguous ways in the abstract texts, so both of the other issues are present in this type of data.

For kNN, there is one hyperparameter, *k*, the number of neighbors to consider. Despite the importance of selecting a good value for *k*, or for selecting hyperparameters more generally, there is not a large body of research literature on this topic. For *k*, we chose to execute a comprehensive grid search for all values of *k* from 1 to *N* on the abstract alone corpus. We optimized for log-loss, a criterion which penalizes errors based on confidence and therefore rewarding conservative prediction (Read et al., 2011). The *k* determined in this fashion is consistent with the optimization of other evaluation measures, such as F_1_-micro (see section 2.4). See the last column of Table 1 for the best value of *k* for each label dimension. For the kNN analyses in our results, we used this optimum *k* for each dimension; it is generally believed that this form of hyperparameter selection is overly optimistic, so the kNN results should be interpreted with this in mind. For more details, see the Supplemental Material.

#### 2.3.3. Sequential minimal optimization

Sequential minimal optimization (SMO) is one of a class of learning algorithms called support vector machines (Platt, 1998). These algorithms have been shown to perform well in text mining applications (Cohen and Hersh, 2005). Support vector machines are a type of hyperplane classifier that seek out hyperplanes that distinguish classes (labels) in the feature space. This is done in such a way that the margin or distance between the boundaries of the classes in the feature space are maximized (so-called maximum margin classification). The methods are called “support vector” machines because a set of vectors lying on the boundaries (the support vectors) are found. Other feature vectors can be changed arbitrarily without changing the classification performance. These methods can be used with non-linear transformations (kernels) but for our corpora dimensions (see section 2.1.1) we can use the linear kernel. The assumption with these methods is that in such a high dimensional space you can find the required hyperplane even without a non-linear transformation.

When using the linear kernel with SMO, there is only one hyperparameter to set, the *complexity*, *C*. This parameter restricts the search space for solutions to the optimization problem; for details, see Platt (1998). We optimized this parameter via a numerical grid search. After extensive work on this, we discovered that the default setting for the WEKA software (*C* = 1) works very well for all dimensions and across all corpora, so this *C* was used for all experiments. This also supports our “off the shelf” approach.

### 2.4. Evaluation metrics

The assessment of algorithm performance in the multi-label problem is substantially more challenging than in the single-label case (Tsoumakas et al., 2010; Madjarov et al., 2012). When an algorithm assigns a set of labels it may assign too few, missing some correct labels that should have been assigned or it may assign too many, adding some irrelevant labels. For any given label, we may easily determine the status, correct or incorrect; but for the entire set of assigned labels the usual case is some labels will be correct, some may be wrong (should not have been assigned), and some that should have been assigned are missed entirely. Evaluating bulk performance, over many labels and many instances is challenging for those reasons as well as the issues related to how the evaluation metrics are to be averaged. Unfortunately there is no single best measure of performance or universally agreed upon set of metrics.

In evaluating our results we used two measures: exact match (also called subset accuracy) and F_1_-micro. Exact match is a very conservative measure of performance; it is simply the percentage of instances which are completely correctly labeled. Any missing, incorrect, or extra labels result in an instance being labeled as incorrect. The measure runs from 0 to 100% and has an obvious interpretation.

F_1_-micro can be formulated as a measure of accuracy that is an average of precision and recall:

$$F_{1}=2\cdot \frac{\text{precision}\cdot \text{recall}}{\text{precision + recall}}$$

where this is the scaled harmonic mean of the two. Precision measures if the labels returned are relevant to the instance, while recall measures the proportion of relevant labels that the algorithm returns out of the total correct labels for an instance. For more details, see Tsoumakas et al. (2010). Missing labels, extra labels, or incorrect labels all reduce the F-score, while correctly chosen labels increase the score. Note that this is the micro-averaged and instance based version of the F measure. This is commonly used when comparisons across data sets are relevant. The best possible F_1_ score is 1 and the worst is 0, but it is not simply a proportion correct, as that concept is not uniquely defined in the multi-label scenario.

The comparison of evaluation metrics across algorithms and across data sets is a source of some debate in the classification literature (Salzberg, 1997; Dietterich, 1998; Demšar, 2006) and very little work has yet been done in the specific case of statistical comparisons for multi-label classifiers. While each fold of the 10-fold validation provides an F_1_-micro, for example, and one can compute a standard error or standard deviation of those 10 values (sometimes reported as the Cross-Validation Standard Error, or CVSE), there are arguments that the CVSE is not, in fact, the basis for any standard confidence interval or any of the usual *t-tests*, as the underlying assumptions for such parametric tests are not fulfilled (Demšar, 2006).

Given that our experiments have a factorial structure, we follow the recommendations of Demšar (2006) and use non-parametric tests, the omnibus Friedman test with a corresponding Nemenyi test as multiple comparison procedure, to analyze our results. These are non-parametric tests similar to the ANOVA in structure. Note that these procedures are completely general and allow the direct comparison of any measure no matter how defined (F_1_-micro, exact match, algorithm run times, etc.) while many other procedures depend critically on the definitions of the measures compared. We specifically evaluate the statistical differences among F_1_-micro measures across algorithms (experiment 1) and corpora (feature spaces) in experiment 2. All statistical thresholds were set at *p* < 0.05.

### 2.5. Software and sources

All of the experiments conducted in this paper were completed using the MEKA software package (meka.sourceforge.net), the multi-label extension of WEKA (www.cs.waikato.ac.nz/ml/weka/). MEKA implements the problem transformation methods and allows the use of WEKA classifiers for the machine learning methods. We used MEKA's BR and LP (called LC in WEKA) problem transformations and WEKA's implementation of NB, kNN (called IBk), and SMO methods. For the problem transformation methods, NB, and SMO we used the default settings; for IBk we used the values of *k* reported above for each data set. Additionally, we used the default Euclidian distance function for kNN and the linear kernel for SMO.

The expert assigned labels for these abstracts have graciously been made available by the BrainMap collaborators. The actual text of the corpora are from PubMed and, as such, are subject to copyright constraints that vary by journal; therefore our data sets cannot be made freely available by the authors of this paper. However, all of the abstracts can be readily downloaded from Pubmed by running a simple Eutils query. The authors will provide a list of MEDLINE abstract numbers or scripts to execute the Eutils query to interested parties. The annotations for this corpus can be accessed through requesting a Collaborative Use License Agreement at the BrainMap website (www.brainmap.org).

## 3. Results

### 3.1. Experiment 1: transformation and algorithm comparison

The focus of the first experiment is on comparisons among methods. We directly compare the various combinations of problem transformation method and machine learning algorithm on the abstract alone corpus for each of the seven CogPO label dimensions. The basic results are presented in Table 2; organized first by transformation and then by learning algorithm within transformation. The rows in the table are the label dimension and the columns represent the results for the three methods SMO, NB, and kNN. The three columns on the left are LP transformed and the three on the right are BR transformed. In each cell, the upper number is F_1_-micro (as a decimal) and the lower number is the exact match percentage. All the values reported in the tables are average estimates obtained from 10-fold cross-validation. Folds were created randomly, with each abstract contributing once to a testing set and nine times to a training set. Balancing the terminologies to ensure that labels in the testing set are always represented in the training set, for example, would likely have improved performance estimates across all the algorithms, but would not have reflected real-world performance or assumptions. The strict maximum F_1_-micro value, for each transformation and label dimension combination is highlighted in boldface, but note that this is not a statistical statement.

**Table 2:** **Performance of SMO, NB, and kNN under the two problem transformation methods, label powerset (LP) and binary relevance (BR)**.

**Dimension**	**Label powerset**			**Binary relevance**		
	**SMO**	**NB**	**kNN**	**SMO**	**NB**	**kNN**
Behavioral domain	**0.413** 29.4%	0.374 25.0%	0.285 14.6%	0.437 24.1%	**0.537** 23.3%	0.350 08.5%
Cognitive paradigm class	**0.460** 43.2%	0.404 37.5%	0.187 17.0%	0.416 28.3%	**0.464** 34.7%	0.262 11.7%
Instruction type	**0.485** 36.1%	0.475 36.5%	0.390 26.8%	0.494 25.9%	**0.538** 23.9%	0.488 20.2%
Response modality	**0.741** 54.2%	0.733 51.0%	0.636 48.2%	0.740 47.4%	**0.744** 49.8%	0.698 41.7%
Response type	**0.704** 51.4%	0.689 51.8%	0.619 41.6%	0.702 44.5%	**0.715** 46.5%	0.656 33.2%
Stimulus modality	0.838 78.1%	**0.842** 78.1%	0.741 68.1%	**0.816** 74.9%	0.814 72.4%	0.768 65.2%
Stimulus type	0.439 30.7%	**0.444** 32.7%	0.317 16.9%	0.387 21.0%	**0.478** 20.6%	0.368 16.5%

All results are based on the abstract alone corpus. Decimals are F*_1_*-micro scores and percentages are exact matches. The strict winner for each transformation-label dimension combination is highlighted. See text for details.

Reviewing the table shows some patterns. Overall performance varies tremendously across label dimensions. This is to be expected, as the complexity of the different dimensions also varies (see Table 1). Less complex dimensions such as stimulus modality are easier to do well on, while more complex dimensions such as stimulus type can do quite poorly. The choice of evaluation metric highlights important points as well: The exact match scores are uniformly greater for the LP transformation then for BR. This is not surprising as the LP transformation treats each unique combination of labels as a distinct entity, so it should be better at exact matches. However, as mentioned above, this leads to a type of overfitting: LP based multi-label classifiers cannot predict novel combinations of labels. Therefore, this increase in performance comes at a price; situations where novel combinations arise frequently will be a problem for this method.

Among the machine learning methods there is no unambiguous single winner, but there is a clear loser. For every label set and under both problem transformations, kNN is always the worst performer. Following the recommendations of Demšar (2006) we used the Friedman test to compare the classifier performance in terms of F_1_-micro values. The LP results showed a significant effect of machine learning method (χ^2^ = 11.14, *df* = 2, *p* = 0.0038) and the results for the BR results were similar (χ^2^ = 12.29, *df* = 2, *p* = 0.0021). This result tells us that the performance of the algorithms are not all the same. Using the Nemenyi (*post-hoc*) test for ranking differences (Demšar, 2006), we can determine which specific methods are different. Under LP, SMO and NB are not significantly different, but both are significantly different from kNN. Under the BR transformation, NB is significantly better than kNN, but there are not other significant differences. It is worth noting that the kNN results are not always so terrible as to be unusable, but the method does sometimes fail dramatically when compared to the other methods.

Comparing performance across transformation methods, each learning algorithm against itself, we see that binary relevance is the clear winner. Both kNN and NB do better under BR than under LP, with kNN always doing better and NB doing better in 6 out of 7 dimensions. SMO does better with LP in 5 out of 7 cases, however, in two of those cases the difference in F_1_-micro is ≤ 0.002. Given the fragile nature of LP compared to BR, this makes a good case for BR as the preferred basic problem transformation method.

Finally, turning to overall best performance, under F_1_-micro the clear algorithm winner is NB (all cases) and BR 6 out of 7 cases (only the stimulus modality labels were better classified using LP). For exact match as a metric, as already mentioned, LP is the better transformation. However, SMO and NB both performed well for some cases and less well for others; NB was the better method for 3 dimensions, SMO for 3, and one dimension (stimulus modality) was a strict tie. See the discussion for more on this.

Upon the suggestion of a reviewer, we explored the actual predicted labels for the different abstracts and label dimensions for the NB-BR method. The goal was to look for label terms which were easily identified (hits), wrongly predicted (false positives), consistently missed (false negatives), or correctly not applied (correct rejections). The results for several of the dimensions are included as heat maps in the Supplemental Material along with discussion. This analysis demonstrated that NB-BR results tend to overpredict labels that were common in the training set, creating false positives, to do well with correct rejections, and miss or fail to predict labels which were more uncommon.

### 3.2. Experiment 2: corpora comparisons

The focus of the second experiment is on the corpora or feature space. The question addressed is whether or not the enhancement of the corpora with more features, such as the MeSH headings and the title text as described above (section 2.1.1) improves classification performance or if similar performance can be achieved with fewer, perhaps more targeted, features (words from titles or MeSH keywords). Given the results of the first experiment, only one representative combination of machine learning method and problem transformation method, NB under BR, was used. The results are in Table 3.

**Table 3:** **Cross-corpora comparison experiment**.

**Dimension**	**Abstract, title, and keyword**	**Abstract alone**	**Title and keyword**	**Title alone**	**Keyword alone**
Behavioral domain	0.534	**0.537**	0.501	0.440	0.448
Cognitive paradigm class	0.464	0.464	**0.471**	0.420	0.394
Instruction type	0.534	**0.538**	0.498	0.488	0.456
Response modality	**0.745**	0.744	0.731	0.710	0.694
Response type	0.706	**0.720**	0.699	0.660	0.662
Stimulus modality	**0.815**	0.814	0.794	0.770	0.805
Stimulus type	**0.496**	0.478	0.470	0.410	0.430

Table presents F_1_-micro values (see text) for naive Bayes under the binary relevance transformation across the five corpora that vary the feature space: words from (1) abstracts, titles, and MeSH keywords; (2) words from abstract text alone; (3) words from both titles and MeSH keywords; (4) title words alone; (5) MeSH keywords alone. Highest F_1_-micro highlighted in boldface; this does not indicate statistical significance. See text for details.

Considering the effect of corpus within each dimension of labels, the keyword alone corpus generally does the worst. The sole exception is for stimulus modality, which is due to the rich MeSH vocabulary for experiments on the visual system. The title alone corpus is not dramatically different from the keyword alone corpus, but there is an apparent, though not statistically significant, performance improvement when these are combined into the title and keyword corpus. (Again this excludes the stimulus modality label dimension). Abstract alone does better than either of the three smaller corpora, and adding everything together into the fullest corpus, abstract, title, and keyword, does not consistently affect performance one way or the other for these data. This is likely due to the abstract text already containing the critical elements of the title or equivalent words. This redundancy also likely explains the performance drop in 3 dimensions (behavioral domain, instruction type, and response type).

The Friedman chi-square on the 7 dimensions by 5 corpora showed a significant effect of corpora (χ^2^ = 22.07, *df* = 4, *p* = 0.0002). The Nemenyi test showed that the worst two corpora, keyword alone and title alone, performed significantly worse than the two best corpora: abstract, title, and keyword; and abstract alone. No other differences were significant.

## 4. Discussion

We present performance characteristics for reproducing expert annotations of a human neuroimaging corpus of manuscripts, using the abstracts of the papers alone and an array of commonly-available multi-label classification techniques. Using an exact match criterion—how often does the method return exactly the labels that the human expert applied to the paper, no more and no less—the label powerset method does the best, in the easiest condition performing above 78%. However, while exact match is easier to interpret, F_1_-micro is a better measure for evaluating performance overall as it does not completely penalize partial matches as complete misses. Using this as a criterion, we conclude that the combination of binary relevance and NB is the best performing combination across the data sets overall.

There is no absolute scale for comparisons of F_1_-micro; there are only relative comparisons across methods and data sets. Its possible values run from 0 to 1, and closer to 1 is better performance, however, this is not a percent correct, nor is it a hit or false alarm rate and must not be interpreted as such. However, to provide some context we examine the results of Trieschnigg et al. (2009). There, six classification systems were compared in terms of their ability to assign MeSH keywords to abstracts, a similar task to ours. In the F_1_-micro scores reported there, one system, the MTI or Medical Text Indexer, obtained a score of 0.4415 and the authors use this as a baseline for comparison with other systems. Note that the MTI is production software that is in actual use. Our hardest label dimension, cognitive paradigm class, in experiment 2 is at about this level of performance and our other label dimensions exceed this (Table 3). This suggests that our classifiers are performing reasonably well, compared to a production system, over all the dimensions on this particular data. We admit, however, that without direct human use studies of such as system as ours, its practical usefulness cannot be determined. (See also the comments on human augmentation below).

In the supplemental materials, we analyze the specific predictions for each instance for two data sets. In the worse performing label dimension, Stimulus Type (F_1_-micro = 0.47), the highest hit rate for a given label in that dimension was 85% (for the label “Letters”); but there was also a 27% false alarm rate for that same label. So if the classifier identifies that “Letters” should be one of the annotations for a given abstract, and given the underlying probabilities of “Letters” in the gold standard annotations, then it would have a percent correct of about 40%; if it identifies that “Letters” should not be one of the annotations, then it would have a performance of about 60%. The performance is similar for the other most common label (“Words”) and worse for the other labels, mostly due to misses. Thus, there is substantial room for improvement across all labels in the worse dimensions, and for specific labels in the dimensions with better F_1_-micro scores. However, following this same type of analysis, if the classifier never guesses “Letters,” then it would be correct 75% of the time (75% of the instances do not have that label), but it would have a miss rate of 100%. Likewise if it always guessed the label “Letters” the hit rate would be 100%, but the false positive rate would also be 100%, leading to an overall performance of only 24% correct, given the frequency of “Letters” as a label in this corpus. In this context, the NB-BR algorithm predictions for that single label appears to strike a reasonable balance between false positives and misses. The overall F_1_-micro for Stimulus Type is of course a combination of performance across the individual labels and not directly predictive of performance on a single label. And we note that most label dimensions have better F_1_-micro scores.

The emphasis in this research has been on the text mining methods, but the nature of the data also affect performance substantially. Turning to this, we see that the performance varied tremendously based across the different label dimensions (compare the rows of Table 3) and performance is less dramatically but significantly affected by changing the feature space (i.e., the corpora; compare the columns). Besides the transformation approaches and classifier algorithms, the structure of the corpus and the structure of the label sets play a role in the ability to perform automated annotation.

Performance across all methods was best for stimulus modality and response modality, which had the fewest labels (5 each), and were among the highest P_min_, or proportion of instances with only a single label. The performance for response type was also notably higher than in the other dimensions, with fewer than 10 labels to choose from and 70% of the instances having only a single label. Performance also dropped off dramatically with either increasing LC_avg_, the average number of labels per instance, or with increasing |*L*|, the number of labels in *L*; the worst performance (Table 3, F_1_-micro, abstract alone column) was for cognitive paradigm class, stimulus type, instruction type, and behavioral domain (in order of increasing performance). These were the dimensions with the largest label sets. Both stimulus type and behavioral domain also had a larger proportion of instances with multiple labels (1 − P_min_), but cognitive paradigm class had a surprisingly large proportion of single-label instances, and yet performed poorly. This suggests that a simpler label structure improves performance.

In Table 4 we show two of our data sets, compared with three other standard data sets used in multi-label classification. These data sets are ordered by complexity, which is usually defined as *N* × |*L*| × *d*; the product of the three relevant set sizes: instances, labels, and features. As shown, relative to other non-biomedical corpora commonly used for multi-label text mining research, our data sets fall toward the lower end of the complexity scale. We include the two extreme complexities for our various sets: “StimModAbs” is the abstract alone corpus with stimulus modality labels, the least complex of our sets; “CogParaAll” is cognitive paradigm labels with the abstract, title, and keyword corpus, the most complex. The other combinations lie between these extremes.

**Table 4:** **Characteristics of several multi-label data sets compared with ours**.

**Name**	**Complexity**	***N***	**|*L*|**	***d***	***d*/*N***	**LC_avg_**	**P_UNIQ_**	**P_max_**
StimModAbs^a^	4,449,705	247	5	3603	14.59	1.15	0.036	0.008
CogParaAll^b^	46,451,808	247	48	3919	15.87	1.13	0.336	0.004
Medical	63,770,490	978	45	1449	1.48	1.25	0.096	0.158
Slashdot	89,777,116	3782	22	1079	0.29	1.18	0.041	0.139
Enron	90,296,206	1702	53	1001	0.59	3.38	0.442	0.096

Values taken from Read et al. (2011); see there for details and sources. For notation, see section 2.1.2 and 2.2. Included are the values for the least and most complex data sets included in this paper.

a Abstract alone corpus; stimulus modality labels.

b Abstract, title, and keyword corpus; cognitive paradigm class labels.

One important feature of the data sets analyzed here is that they are unusually small (in terms of instances) and large (in terms of features) compared to many other standard data sets (compare *d* and *N* columns, also presented as a ratio in the *d*/*N* column). We expect in ongoing research to make use of larger pools of data from BrainMap, or other databases, which lead to complexities greater than 10^8^ or an order of magnitude larger than the standard test sets in Table 4. If the dictionaries do not dramatically expand, this leads to *d*/*N* ratios closer to 1. Note that there are test sets in use, such as the MEDLINE baseline distributions (www.nlm.nih.gov/bsd/licensee/baseline.html) or OHSUMED (ir.ohsu.edu/ohsumed/ohsumed.html), among others, that are comparable with or exceed these larger sizes. However, the data sets derived from the scientific literature will continue to have a particularly rich text feature space and therefore large *d* values.

The number of features is at least 3603 for all corpora using abstract text, and only 247 instances. The ability to identify synonyms or reduce this *d* through other means may improve performance, which is within the scope of future work. The Colorado Richly Annotated Full Text Corpus (CRAFT; bionlp-corpora.sourceforge.net/CRAFT/index.shtml) is a counter-example, including only 67 papers originally, but that includes full text, and a substantial effort at detailed syntactic annotation and concept identification, with a final count of 793,627 tokens and many thousand annotations (Bada et al., 2012; Verspoor et al., 2012). Their annotations were focused on syntactic parsing of example genetic literature, and as such, the annotations were parts of speech and similar tags, rather than our goal of identifying multiple labels from different possible dimensions specific to neuroimaging experiments. Their parsing results are promising, however, for future more sophisticated applications to this domain of biomedical literature text and concept mining.

Note also that our data sets have labels from specific non-interchangeable dimensions; they are not simply a single bag of multi-label possibilities. Thus, as repeatedly noted above, they are not directly comparable to the common test cases. While the number of labels, LC_avg_, and other measures are within the range used in other corpora, our data have relatively low complexity due to the small number of instances (247), an order of magnitude less than most other data sets used in this work. See Madjarov et al. (2012) and Read et al. (2011) for summary statistics on several additional comparable data sets.

It is worth noting that in the MeSH markup task in Trieschnigg et al., the test set was 1000 abstracts with a label set of 3951 MeSH terms; two orders of magnitude larger than our largest label dimension. Comparing those results with ours suggests that F_1_-micro may be a function of the number of labels |*L*| or possibly some scaled version of this. Unfortunately, neither Trieschnigg et al. (2009) nor Trieschnigg (2010) provides an exact number for the size of the training sets used for their kNN classifier, so we cannot make that comparison. However, they appear to have used large sets, with “at most” 1000 citations per MeSH term (Trieschnigg et al., 2009). It is important to contrast this with the number of training/testing instances we used which was 247 total. This suggests that relatively high performance may be achieved with very limited data (instances) given the richness of the feature space derived from abstract text.

One of the primary goals of this project is to develop text mining methods that can improve PubMed searches. This leads to an emphasis on abstracts. The expert annotators for this corpus used the full-text of the papers to make their label determinations; thus, they had access to more information than was contained in the input to the machine learning algorithms. We have a second project underway with a number of expert curators attempting this task on a subset of the abstracts; they may do better, they may do as well, or worse than the blind statistical approaches. Some of the variation in human performance is expected to be quite informative about which dimensions, and which terms, are more easily identified by experts and which are not. Those data are not yet available and are planned for a second paper that addresses the human aspects of these efforts in more detail. A quick interaction with several experts identified that most abstracts contain enough information for them to guess one or two of the annotations quite accurately (e.g., the paradigm type, stimulus modality, etc.) but not to get an exact match, though we do not yet have robust performance estimates.

For instance, the cognitive paradigm class label “go/no-go” implies a task that has the stimulus modality label “visual,” response modality “hand,” and response type “button press.” This implication is not logically necessary (it is possible that it be otherwise) but for the papers in the BrainMap database, this implication is effectively certain. Additionally, there are logically necessary dependencies; for example, a “flashing checkerboard” (stimulus type) is necessarily presented to the “visual” stimulus modality. Expert annotators use both of these types of dependency knowledge in their label assignment task. None of the methods tested here use this information explicitly. There are more much complex approaches, some of which include statistical and logical dependency information. We are in the process of developing a new algorithm (constrained hierarchical Bayes) that is the topic of other presentations (Chakrabarti et al., 2013). We expect that they may lead to improved performance by incorporating dependencies of the type that humans use to reason.

A challenge for these techniques is the flexibility to handle new instances as they arise in new data; in the neuroimaging literature, new experimental paradigms arise frequently, and the CogPO terminology is expected to grow. This growth will be (1) in the addition of new terms for novel paradigms and (2) in the introduction of more precise terms as the granularity of the system moves from coarse to fine grained. BrainMap itself has already undergone several additions to the original term lists prior to the development of CogPO, with old terms being refined into several new terms. Each time new terms were included, it required a re-labeling of many experiments, to make sure their annotations are consistent with the updated label lists. This process will continue as research in these areas continues, cognitive experiments become ever more refined, new subdivisions of behavioral domains or cognitive processes come into vogue, and so on.

This is a problem for the label powerset transformation method; it is fragile with respect to label combinations. It cannot correctly label an instance which has a novel combination of annotations without retraining its underlying classifier on explicit examples of the new label combination. Thus, while this method had an advantage over binary relevance in the exact match measures, given the issues with extending the label powerset approach to the ever-expanding scientific literature—with the constant influx of new label combinations—its modest advantage over binary relevance is not sufficient to recommend it, at least not as a singular solution. However, binary relevance has the reverse problem, it cannot specifically model combinations of labels that carry the contingent or conditional information discussed above, and so its advantage in being less fragile is somewhat offset by this loss. While binary relevance is the better method given the present constraints, we anticipate future methods that combine the benefits and offset the losses of each of these methods when used as pure methods.

An additional complexity is that the original annotations for stimulus type, instructions, and response were made for each paper based on the experimental conditions. Each experiment reported in a paper is made up of conditions, which are generally (though not always) distinguished by some difference in the stimulus, instructions given, or responses made by the subject. The comparison of brain imaging results across different conditions tends to be the basis for the results presented in the paper. In our case, the annotations on the abstracts are provided as a set, without taking into account which combination of stimulus, response, and instructions formed an experimental condition. A different line of study would consider the stimulus/response/instruction combinations per condition as the labels to be predicted, and determine whether these algorithms improve in performance. There are many nuances to this problem. For instance, one example which would require through exploration would be bootstrapping an identification of stimulus and response to predict the likely instruction label. There are many others. Insofar as it is the combination of experimental conditions that identifies the cognitive process under study, the ability to identify the conditions might be key to classifying the abstracts as being “working memory” or “attention” studies; the more granular level of description, such as the use of a particular stimulus or instruction set, however, can also constrain the relevant cognitive circuitry and the ability to identify relevant abstracts for meta-analysis or other purposes. While an analysis that treats the relevant stimulus, response, and instruction combinations as label sets to be predicted is outside the scope of this original approach, it is definitely worth considering as a future analysis.

The structure of ontologies for biomedical annotation certainly requires some consideration. As noted in Bada and Hunter (2011), ontologies for full-text, generic biomedical annotation should meet a number of requirements. CogPO meets several of these requirements, being a mid-level ontology with defined terminology and built on the widely-used Basic Foundational Ontology (BFO; www.ifomis.org/bfo), but it falls short of having richly defined relationships, logically constrained definitions that are unambiguous, and its representation of synonyms and acceptable alternative terms is sorely lacking. There need to be many levels between specific terms (or synonym classes) and high level concepts that are very abstract; this allows for retrieving similar results or being able to generalize to related terms. This is an area that appears open to formal analysis, but to date this analysis is lacking.

These richly-defined relationships and definitions specified in formal logic are less relevant for the kinds of classifiers we implemented in this work; we are using the labels as standard terms without any of the logical constraints or relationships defined across ontological classes. The labels here are used more as a controlled vocabulary than as an ontology *per se*. But the ability to identify alternative forms (synonyms) of labels would certainly improve performance, as would having a deeper hierarchy, with general classes broken into subclasses. For example, identifying that “Auditory Oddball” and “Spatial Oddball” are both “Oddball” paradigm classes, would allow the label “Oddball” to be identified without being completely correct, as a generalization of the finest-grained correct label. Incorporating this level of performance as a recommended term could facilitate the human annotator's job, as they now have a good reason to believe the Paradigm Class is an Oddball and only need to consider a more limited number of subclasses as potential annotations. It is worth mentioning that this conditionalization can be exploited by machine learning algorithms (Jones et al., 2013).

While machine-learning and text mining techniques have been applied in various biomedical domains to facilitate annotation or tagging, applications to human neuroimaging are rare, and the application to replicating expert-provided annotations regarding cognitive experimental details is available only through databases such as BrainMap or the derived Brede database (neuro.imm.dtu.dk/services/brededatabase/). The Neurosynth project www.neurosynth.org; Yarkoni et al., 2011) is an innovative text-mining effort based on full-text analysis of many neuroimaging papers, tagging papers and their imaging results with the most common words in the text. This allows searching the database of papers by brain region, cognitive paradigm, or other common technical terms. To date these attempts have focused on repetition of words for tagging, rather than identifying what the details of the experiments are, and thus what the results of the experiment might indicate. It is important to note that the classifiers developed on abstracts may not generalize directly, without any change; as noted in Cohen et al. (2010), the linguistic content of abstracts is different from the content and structure of the full text of the document. As full text documents which are annotated with standardized terms from CogPO or other ontologies for human neuroimaging experiments become more plentiful, it is expected that the use of the Methods sections from those papers will lead to better performance in automatically annotating experimental designs. However, at the moment there are no readily accessible collections of the methods, or other sections, of papers making direct experimentation impossible. As more full-text is curated, it will be possible to extract other sections of technical papers for analysis. We expect the processes here to generalize, albeit with different underlying dictionaries.

The ultimate goal of these text mining approaches is to provide automated annotations of functional neuroimaging literature, to enhance the utility of neuroimaging databases, to increase the speed of populating those databases, and to improve the accuracy and specificity of literature searches. The classifiers under consideration in this paper are only part of the solution. First, the abstracts we are working with were already identified by human experts as fMRI or PET human neuroimaging papers. Identifying from PubMed which papers are human cognitive neuroscience papers and which are not can be done to a certain extent through careful PubMed querying, but not yet with perfect sensitivity and specificity. Also, in this analysis we do not distinguish between experiments and papers (which often contain multiple experiments) as we are using the abstract text only and many abstracts do not provide clear demarcation between experiments. Currently, only expert human annotation can link the specific experimental design elements with specific experiments in a paper. We expect that the methods here will readily extend to other sections of papers, allowing full classification of individual experiments. Using a combination of binary relevance and NB gives a fairly good guess for several of the CogPO dimensions based just on the language used. Without performance improvement, the classifications for other dimensions using these methods would have to be considered suggestions to be confirmed, denied, or added to based on the human expert's judgment. Methods that link across label dimensions may improve performance, e.g., leveraging knowledge about the combinations of stimulus, response, and instructions that define certain cognitive paradigms, would be needed to filter papers for a focused meta-analysis.

Beyond identification of the experimental methods and details, papers contain results in the form of numbers, tables, and figures. The encoding of this information in a form appropriate for storage in a database is currently a human task. Obviously, papers with multiple experiments only complicate this problem as well. Both of these tasks must be done in order to carry out appropriate meta-analyses.

Given our motivating problem of facilitating curation—automatically identifying the appropriate annotations for a neuroimaging experiment—the performance of fairly basic classifiers indicates that some of the annotations can be identified quite accurately using these methods. We envision the application of iterations, preferably a learning algorithm which can suggest papers for a meta-analysis, and as papers are accepted or rejected by the investigator, the algorithm performance improves.

### Conflict of interest statement

The authors declare that the research was conducted in the absence of any commercial or financial relationships that could be construed as a potential conflict of interest.
