# Supplementary material for: Automated annotation of functional imaging experiments via multi-label classification
Source: Front Neurosci. 2013 Dec 16;7:240. doi: 10.3389/fnins.2013.00240 (PMC3864256; doi:10.3389/fnins.2013.00240)

Supplemental Materials for: Turner, Chakrabarti, Jones, Xu, Fox, Luger, Laird, and Turner, "Automated annotation of functional imaging experiments via multi-label classification", **Frontiers in Neuroscience, Brain Imaging Methods**.

Section 3.1 – Experiment 1 Supplement

It is informative to consider in more detail the specific errors that the classification algorithm makes. Here we review in more detail the predicted versus actual annotations for response modality, as that was one of the best overall performances, and for stimulus type, as that was one of the worst performances using the naïve Bayes, binary relevance (NB-BR) classifier on the abstract alone corpus. In order to get predictions for each abstract, the classifier was trained using a leave-one-out model (as opposed to the 10-fold cross-validation used in the main paper) and then predicted the labels for the abstract which had been held out. In each case, the classifier tended to over-predict the more common terms, and generally failed to predict the rarer labels.

In order to perform this analysis, we used the software tools developed in Chakrabarti, *et al.* (2013) which provided the data in a format amenable to this analysis. This software allows a NB-BR analysis of the same type as that performed in the main manuscript.

Label Frequencies

When considering label frequency, there are two ways to order the labels. The first considers labels independent of the label sets applied to each instance. So a given label may appear in 50% of instances, say, where in some of these the label appears alone, in others it appears in combination with one other label, in combination with two other labels, and so on. We call this the *independent ordering* of the labels. The second way to consider ordering is by the frequency of the label combinations or whole sets of labels. In this ordering, the unique label sets are ordered by frequency; we call this the *combination ordering*. Note that, importantly for the discussion that follows, this ordering will have sets of labels that contain a single label and this set of a single label may be the most common by this ordering; and yet the label is simultaneously not the most common in the independent ordering. Note also that the relative frequencies in combination ordering add up to 1.0 (100%) while the independent ordering does not follow this constraint.

Response Modality Labels

Response modality had 5 possible labels in this corpus (Foot, Hand, Ocular, Oral, and None) of which Hand and None were used in 70% and 52% of the corpus respectively, while Foot, Ocular, and Oral were each used in fewer than 2% of the data (independent ordering). The most frequent label sets (combination ordering) were {Hand} with 44% of instances; {Hand, None} with 24% of instances; and {None} with 22% of instances. There were nine unique combinations (as can be estimated from Table 1 in the manuscript), of which two were only used once.

In **Supplemental Figure 1**, we provide a heat map for the performance of the NB-BR classifier over the 247 instances and 5 labels, showing the hits, correct rejections, misses, and false alarms for each

abstract. In **Supplemental Table 1**, we include the summary hit, miss, correct rejection, and false alarm rate for each label, with the caveat that those measures should not be interpreted as they are in a single-label case. The LC_{avg} in the original dataset was 1.15, and the LC_{avg} in the predicted labels was 1.17. The three labels which were rarely available in the dataset were never predicted (no red or orange in the foot, oral, and ocular columns of the figure), and thus contributed only misses and correct rejections to the results. Hand and None were predicted for 73% and 44% of the abstracts, respectively, with the combination (Hand, None) predicted for 18% of the abstracts.

Stimulus Type Labels

Stimulus type, in contrast, had 17 possible labels used in this dimension. See **Supplemental Figure 2**.

Considering the independent ordering of label frequencies, the most common label was Words, applied to 30% of the abstracts, followed by Letters, applied to 24%. There were some labels that were applied only rarely: there were 4 labels that applied to only one instance each (Asian Characters, Eyepuffs, Odor, and Pseudowords); and two labels that were used on three instances each (Noise and Symbols). All other labels were used on at least 4 instances.

Considering the label set frequencies, in combination ordering, the most common sets were:

- {Letters} 13%
- {None} 12%
- {Words} 10%
- {Faces} 7%
- {Pictures} 6%
- {Words, None} 4%

where the percentage in the table is the number of instances with that label set. Thus, 48% of the instances in the data had only single labels taken from Letters, None, Words, Faces, and Pictures; and 52% of the data is either so labeled or has the double label {Words, None}. The remaining 48% uses the remaining labels in various combinations (including singleton label sets). There were 61 unique label sets (as can be estimated from **Table 1** in the manuscript), of which 31 were used only once, which would account for the loss in performance using the LP problem transformation.

Using the NB-BR classifier as above for these labels, the LC_{avg} for the complete dataset was 1.49, and for the predicted labels it was also 1.49. In **Supplemental Figure 2**, we provide a heat map for the classifier output, with 247 abstracts in the rows and the 17 possible labels as the columns. Again, the labels with the fewest instances in the training set were rarely if ever predicted, while the most common labels were predicted more than they occurred in the training set. The label Words was predicted for 42% of the abstracts, while Letters was predicted for 40%, and the combination of {Words, None} was predicted for only 3% of the abstracts.

Supplemental Figure 1. Heat map of the predicted Response Modality labels for the 247 abstracts, in no particular order, along the y axis. Colors: dark blue = correct rejection; light blue = miss, i.e. label should be assigned, but was not; light orange = false positive; deep red = hit, i.e. correctly predicted label.

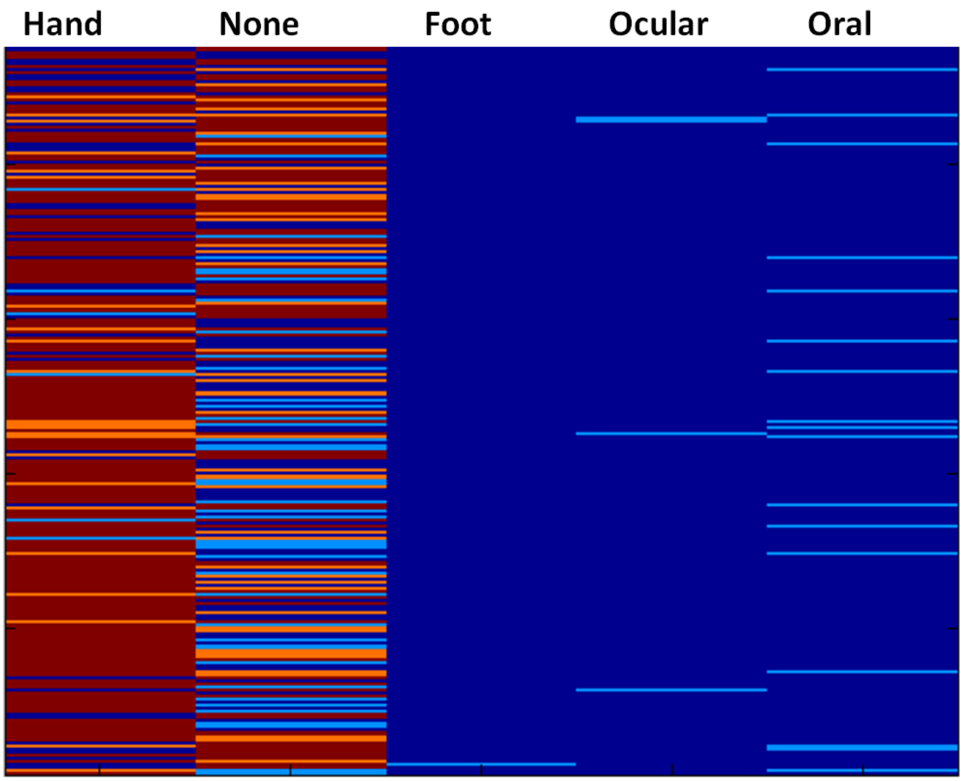

Supplemental Table 1. Summaries of Supplemental Figure 1. Frequency is the proportion of abstracts that included that label; hits, misses, false alarms and correct rejections are the relevant proportions.

Label	**Frequency**	**Hits**	**Misses**	**False Alarms**	**Correct Rejections**
Hand	0.692	0.959	0.041	0.329	0.671
None	0.514	0.677	0.323	0.392	0.608
Foot	0.004	0	1	0	1
Ocular	0.016	0	1	0	1
Oral	0.081	0	1	0	1

Supplemental Figure 2. Heat map of the predicted Stimulus Type labels for the 247 abstracts, in no particular order along the y axis. Colors: dark blue = correct rejection; light blue = miss, i.e. label should be assigned, but was not; light orange = false positive; deep red = hit, i.e. correctly predicted label.

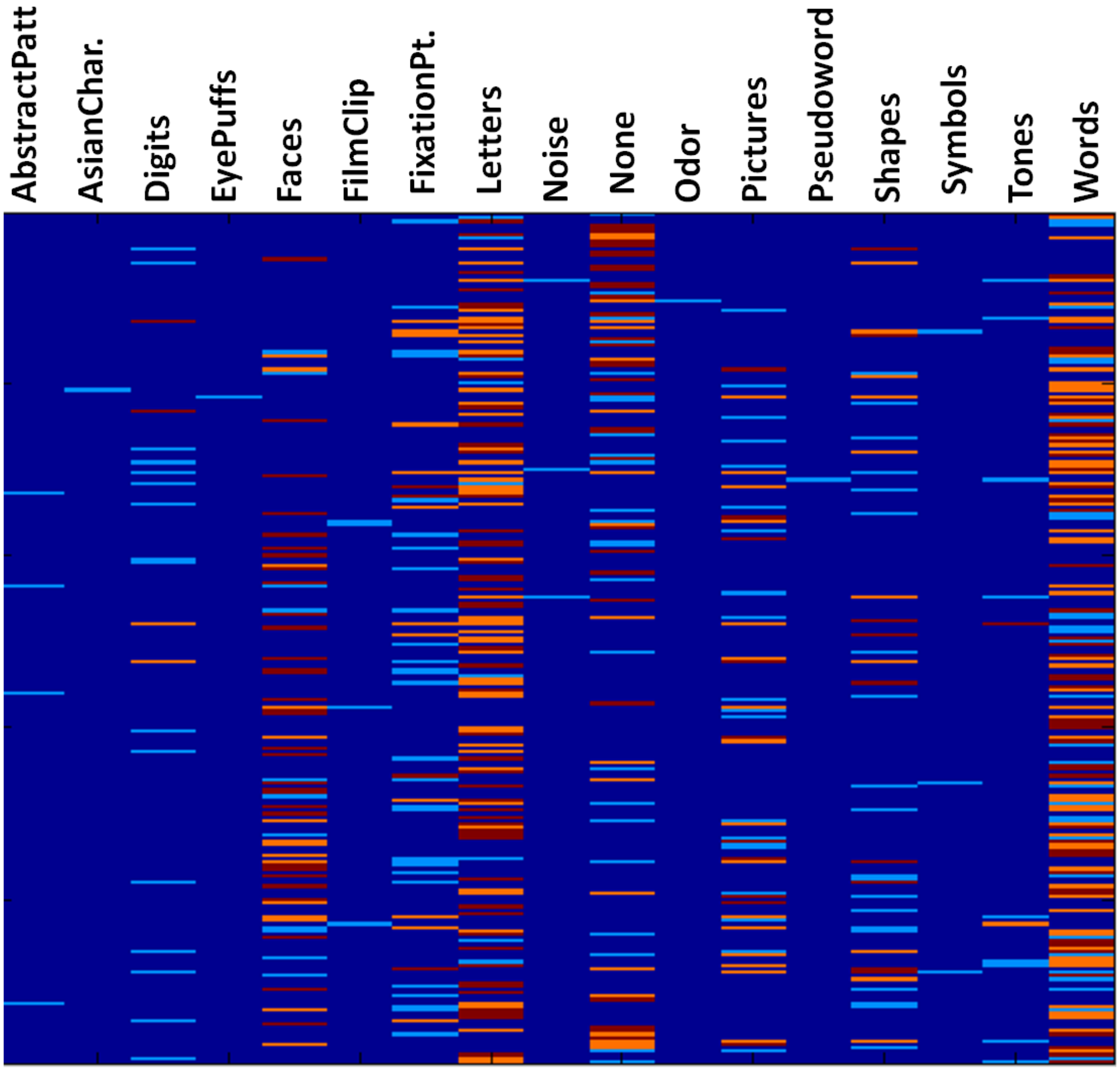

Supplemental Table 2. Summaries of Supplemental Figure 2. Frequency is the proportion of abstracts that included that label; hits, misses, false alarms and correct rejections are the relevant proportions.

Label	Frequency	Hits	Misses	False Alarms	Correct Rejections
**AbstractPatterns**	0.016	0	1	0	1
**AsianCharacters**	0.004	0	1	0	1
**Digits**	0.073	0.111	0.889	0.008	0.991
**EyePuffs**	0.004	0	1	0	1
**Faces**	0.174	0.744	0.256	0.073	0.926
**FilmClip**	0.016	0	1	0	1
**FixationPoint**	0.129	0.125	0.875	0.056	0.944
**Letters**	0.239	0.847	0.153	0.266	0.734
**Noise**	0.012	0	1	0	1
**None**	0.223	0.564	0.436	0.099	0.901
**Odor**	0.004	0	1	0	1
**Pictures**	0.121	0.333	0.667	0.074	0.926
**Pseudowords**	0.004	0	1	0	1
**Shapes**	0.117	0.310	0.689	0.046	0.954
**Symbols**	0.012	0	1	0	1
**Tones**	0.040	0.100	0.900	0.004	0.996
**Words**	0.304	0.613	0.387	0.337	0.663

Section 2.3.2 – Supplement to Hyper-Parameter Setting (*k* in kNN)

As mentioned in the main paper text, there is no completely agreed upon method of selecting and setting hyper-parameters for machine learning algorithms. In the cases of NB and SMO, we chose to use the off-the-shelf defaults in WEKA. For kNN, the parameter *k* has no default setting and must be chosen. We selected our values of *k* by minimizing the log-loss performance on the plain corpus for each label set (Read et al., 2011). Our *k* selection algorithm was to chose the *k* that minimizes log-loss for the labels; in the case where several values of *k* give the same minimum value we chose the smallest *k*. One of the reviewers asked for us to provide plots of the log-loss performance of kNN in the hyper-parameter setting. These plots appear as supplemental figures 3-9, below.

Supplemental Figure 3.

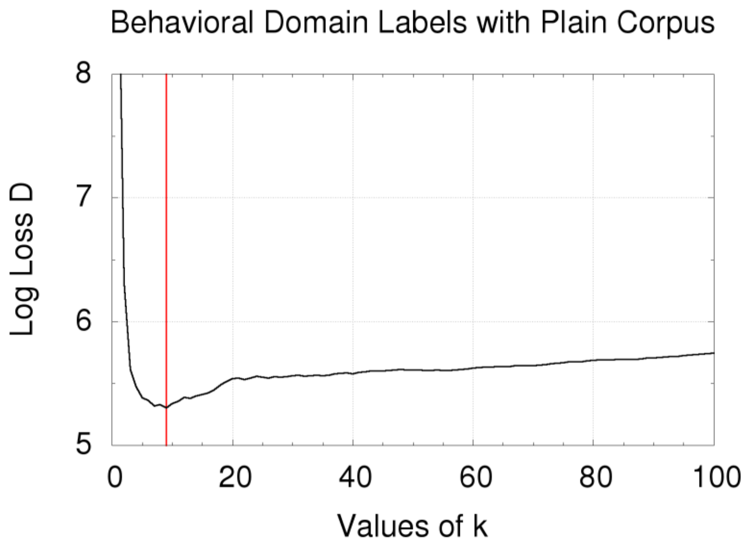

Supplemental Figure 4.

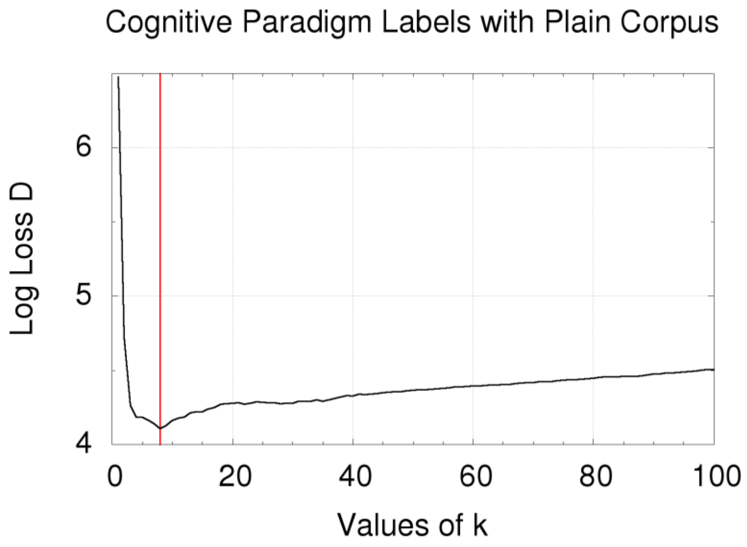

Supplemental Figure 5.

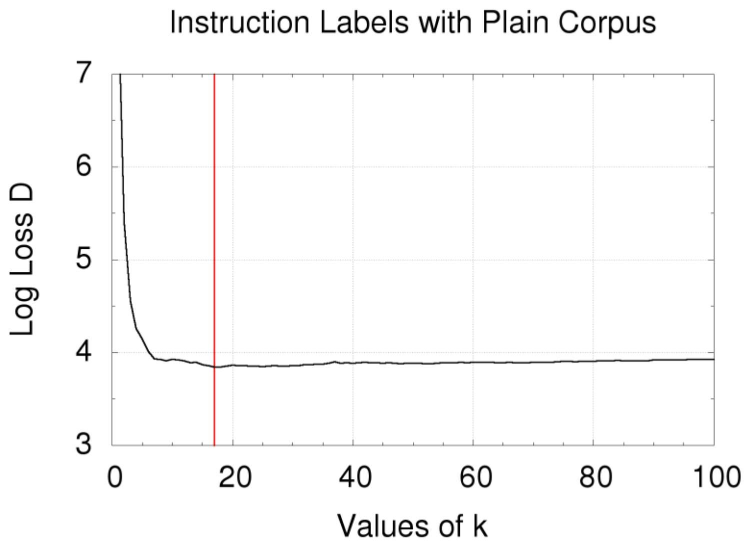

Supplemental Figure 6.

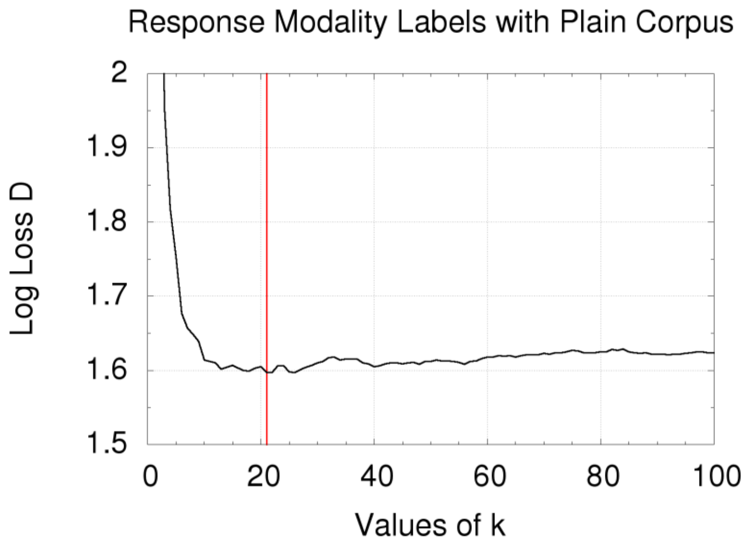

Supplemental Figure 7.

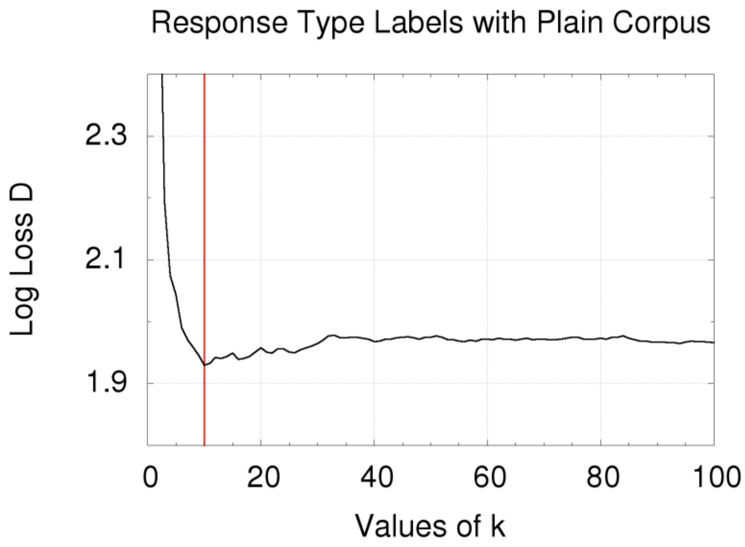

Supplemental Figure 8.

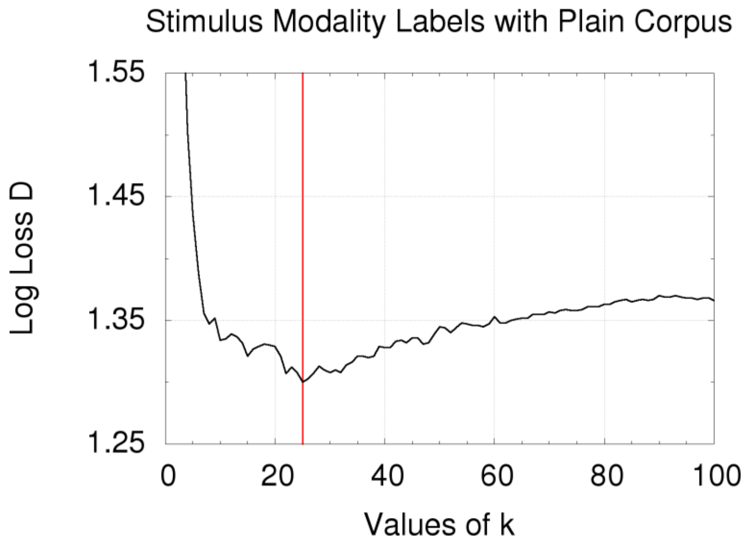

Supplemental Figure 9.

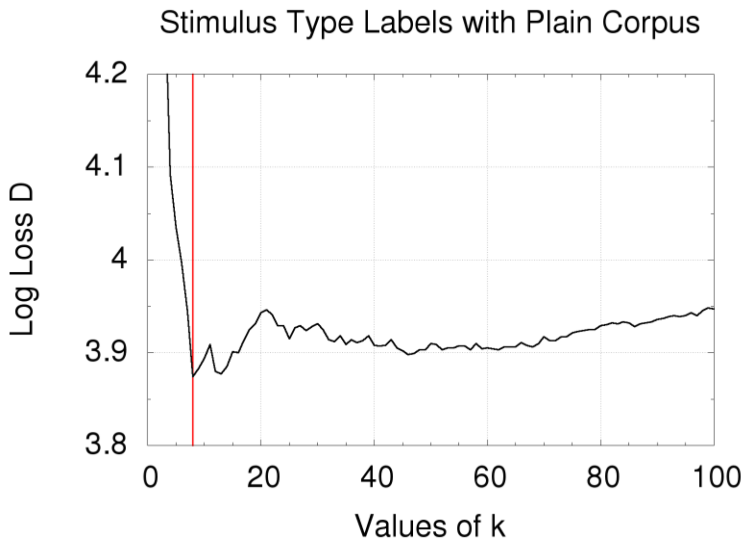

Supplement: Supplementary file 1 [file Presentation1.PDF]
